# In Search of “Just Right”: The Challenge of Regulating Arsenic in Rice

**DOI:** 10.1289/ehp.123-A16

**Published:** 2015-01-01

**Authors:** Charles W. Schmidt

**Affiliations:** **Charles W. Schmidt**, MS, an award-winning science writer from Portland, ME, has written for *Discover Magazine*, *Science*, and *Nature Medicine*.

Rice, a dietary staple for millions of people around the world, is often contaminated with arsenic, a naturally occurring element in soils that can cause cancer and other health effects.^1^ Although other foods also contain arsenic, rice is unusually efficient at absorbing this element from soil; it can absorb up to 10 times more arsenic than other crops, such as wheat.^2^ Moreover, rice flour and syrup are used in many processed foods, including baby foods, so exposures aren’t limited to people eating the grain itself. It’s estimated that 95% of the average arsenic intake among Europeans comes from food, and half of that comes from rice and rice products.^3^ And in areas with high levels of arsenic in well water, the exposures via water and rice add up to a toxic double whammy.^3^

Mounting worries over arsenic in rice are now prompting calls for regulation. “We need to set strict standards for rice that will be meaningful in terms of reducing arsenic exposure through the diet,” says Andrew Meharg, a professor of biological sciences at Queens University Belfast in Ireland. “This is imperative to protect people with high rice consumption, including virtually all children, people living in South Asia, and those who eat a lot of rice for health reasons, such as gluten intolerance.”

**Figure:**
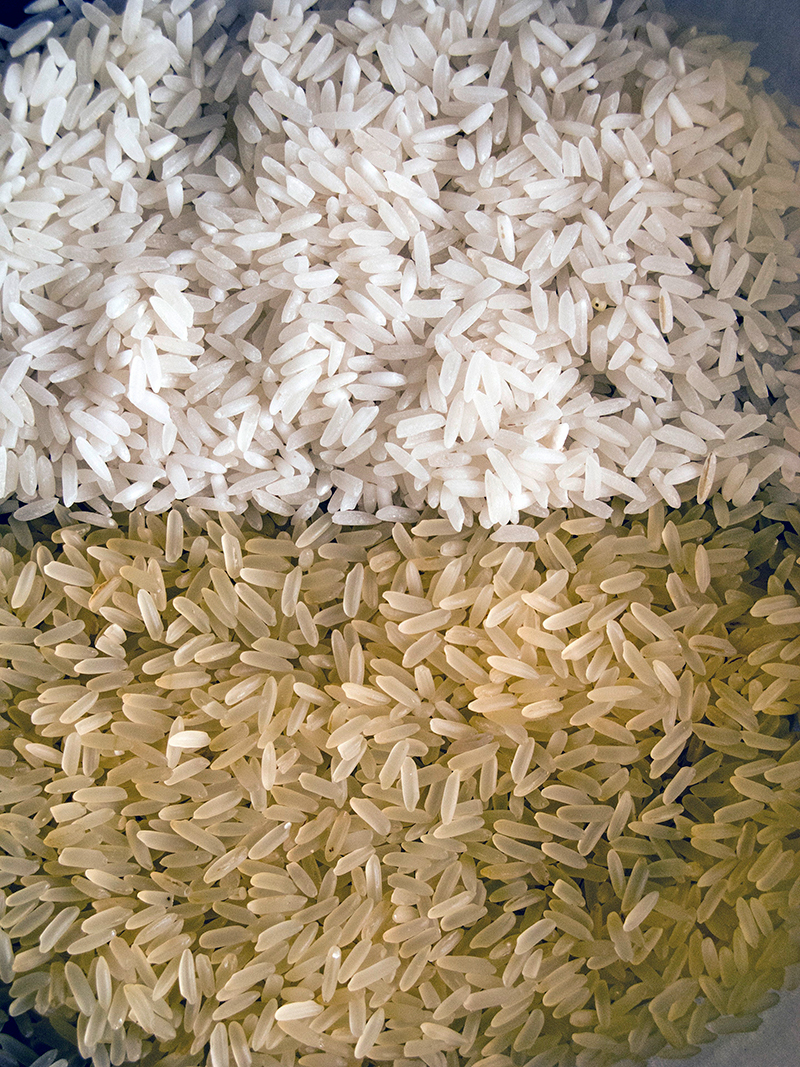
A regulation that’s too high may not adequately protect health, and a regulation that’s too low could be infeasible for producers to achieve. © 145/Steve Outram/Ocean/Corbis

But regulating a naturally occurring element in such a widely eaten food is no easy task. Arsenic levels can vary widely in rice from different countries and states, and among different rice cultivars, according to Aaron Barchowski, a professor of environmental and occupational health at the University of Pittsburgh. This raises difficult questions about how a regulated standard could be monitored and enforced.

## Assessing the Threat

The U.S. Environmental Protection Agency (EPA) currently designates arsenic as a nonthreshold carcinogen, meaning that any dose, no matter how small, carries some cancer risk.^4^ Some scientists don’t agree—they say doses below a certain threshold won’t cause cancer, a debate that has yet to be resolved.^5^

In another area of uncertainty, Michael Crupain, director of food safety testing at the testing group Consumer Reports (CR), notes that scientists have not documented elevated rates of bladder and lung cancer—the more lethal malignancies with which arsenic in well water is most often associated^6^—in countries where rice is commonly eaten in large amounts. “Carefully designed studies investigating this question need to be conducted,” he says.

However, studies also reveal associations between arsenic and numerous health effects, including cardiovascular disease,^7^ lung disease,^8^ and impaired cognitive function,^9^ among many others. Barchowski explains that arsenic in small amounts stresses cells, making them prone to maladaptive reactions that promote disease over time.

Children in particular appear to be uniquely sensitive to low doses of arsenic.^10^ Investigators in both rural Bangladesh and the United States, for instance, have shown that fetal exposure to arsenic is associated with respiratory infections and diarrhea during infancy and early childhood.^10^^,^^11^^,^^12^ Moreover, cross-sectional epidemiological studies in Bangladesh and in Taiwan have connected early arsenic exposures with neurobehavioral problems in school children and adolescents.^1^

While people can be assumed to drink water from the same well on a consistent basis, the amounts of arsenic ingested from food can be far more difficult to quantify, according to Habibul Ahsan, a professor of health studies, medicine, and human genetics at the University of Chicago. Dietary effects vary by whether the arsenic is organic or inorganic (the latter being more toxic) and by the amounts of arsenic in a given food, he says, and the absorption of arsenic from the gut into the bloodstream also varies by food type.

Barchowsky points out that rice and rice products contain many nutrients—for example, B vitamins and selenium—that can protect against the toxic effects of arsenic.^13^^,^^14^ “This greatly complicates assessing the real risk of eating rice with arsenic in it and is a point that is not raised often enough,” he says. “Eating a healthy balanced diet reduces risk.”

## Moving toward a Standard

The U.S. Food and Drug Administration (FDA) has spent years grappling with the issue of arsenic in rice. It’s currently in the midst of a health risk assessment that officials say would help form the scientific basis for any future regulation. The agency currently advises parents to consider diversifying the grains they feed their infants and toddlers, and encourages all consumers to read products labels for rice-based ingredients, and to consume a variety of grains.^15^

In the meantime, Codex Alimentarius, a body coordinated by the Food and Agriculture Organization of the United Nations (FAO) and the World Health Organization (WHO) that develops international food standards, has proposed a maximum level of 0.2 mg/kg for inorganic arsenic specifically in white (or polished) rice.^16^ However, Codex recommendations are nonbinding—countries can adopt and enforce them at their own discretion. And some critics say the proposed rice standard, which was announced in July 2014, isn’t protective enough.

White rice makes up 79% of the international market.^16^ but the highest arsenic levels are consistently found in brown rice. That’s because rice kernels concentrate arsenic in the thin outer layer that gives brown rice its color and is removed to produce white rice.^17^ Organic brown rice syrup, a popular sweetener often touted as a healthier alternative to high-fructose corn syrup,^18^ has been found to contain a similar range of arsenic levels as brown rice grain.^19^

According to Angelika Tritscher, the coordinator for risk assessment and management with the WHO Department of Food Safety in Geneva, Codex attempted to set a standard for brown rice and proposed a value of 0.4 mg/kg, but could not reach agreement because of insufficient data on arsenic levels in brown rice occurring globally. The discussion of such a standard will be continued at the next meeting of the Codex Committee on Contaminants in food in March 2015.

## Finding the Right Balance

Both the EPA and the WHO have adopted maximum limits of 10 µg/L for inorganic arsenic in drinking water.^20^^,^^21^ However, most countries do not currently regulate arsenic in rice. The European Union—which sets centralized food safety standards for its member countries—has come out in favor of the Codex white rice standard, but it has yet to endorse it as law.^22^ According to Meharg, the European Union also plans to adopt a value of 0.1 mg/kg that would be specific to inorganic arsenic in rice-based baby food.

Codex based its white rice standard on sampling data collected from ten countries in Europe, North America, and Asia. The intent was to set a level that would reduce arsenic exposure but that wouldn’t be so low that most countries wouldn’t be able to meet it. Otherwise, Tritscher explains, “The limit [would have been] hypothetical, with no practical relevance.” And the essential requirement for any arsenic regulation, she says, is that it can be enforced. Codex is developing additional guidance to help producers meet the standard.

Maximum inorganic arsenic levels in the submitted samples ranged between 0.16 and 1.8 mg/kg, but the mean values were all below 0.2 mg/kg.^23^ Thus, the 0.2-mg/kg value was selected in part because of its feasibility, with a relatively low exceedance rate of 2%. The official language used by Codex to describe the standard is that it is “a maximum level deemed to be as low as reasonably achievable.”^24^

That the standard is achievable is borne out by other sampling data. In 2012, for instance, CR tested 223 samples of rice and rice products purchased in the United States and found nearly all of them were below 0.2 mg/kg.^25^ The next year the FDA published an analysis of more than 1,300 samples^26^ and concluded that “the amount of detectable arsenic is too low in the rice and rice product samples to cause any immediate or short-term adverse health effects.”^15^ The next step, the agency said, is to learn more about the impact of long-term, low-dose exposures.^15^

## Not Quite There

Meanwhile, the proposed Codex standard has come under attack by those who say it has no basis in health risk assessment. Meharg, for instance, champions a lower value of 0.1 mg/kg for all rice products, and an even lower value for products targeted at young children and babies, where he believes that 0.05 mg/kg is readily achievable. And Consumers Union (CU), the policy and advocacy arm of CR, has called on the FDA to adopt a standard of 0.12 mg/kg for both white and brown rice and rice products.^27^

Michael Klein, a spokesman for the Arlington, Virginia–based USA Rice Federation, agrees with how the Codex standard was derived. Setting it too low, he says, would have “wiped out the rice industries in some countries.”

But Meharg disagrees. “The standard should protect people’s lives and health, and as it is now, it doesn’t do that,” he says. “It gives no incentive to change agricultural practices or processing, and it justifies the status quo.”

According to Crupain, CU’s proposed standard of 0.12 mg/kg is based on a health risk assessment that assumes a nonthreshold dose response for cancer. “While it isn’t a threshold for safety, it does provide a reasonable and feasible starting place for a standard,” he says. He also says data from the FDA and CR indicate almost 90% of white rice and 28% of brown rice in the United States could meet this standard.

## Other Solutions

The goal of reducing arsenic exposures from rice doesn’t lend itself to easy solutions. In a new report CR recommends that people limit their weekly rice consumption to just over 1 cup (uncooked) of rice produced in areas with lower detected levels of arsenic—specifically basmati rice from India, Pakistan, and California, and sushi rice from the United States.^28^ For rice from areas with higher arsenic levels, CR recommends limiting consumption to about half that amount for adults and one-quarter that amount for children.^28^

Scientists are also exploring other options that include breeding arsenic resistance into rice plants—some rice varieties absorb less arsenic than others, and studies so far suggest these traits can be successfully cross-bred into progeny.^29^ Soils can be inoculated with microbes that act to slow arsenic uptake through the roots, and likewise, rice can be genetically engineered in ways that prevent arsenic uptake, adds Barry Rosen, a professor of cellular biology and pharmacology at Florida International University. Rosen recently developed a transgenic rice plant that can methylate inorganic arsenic into less toxic organic forms.^30^ He says a commercially viable cultivar is still decades away.

Asked if genetic engineering poses acceptable solutions, Tritscher says, “We need to keep an open mind. … I would not exclude any reasonable option for improving the situation of arsenic in rice. But the safety of any new technology or agricultural procedure needs to be assessed first.”

Klein of the Rice Association is more skeptical. “Posed with a choice between [genetically engineered] rice and rice with arsenic in it, consumers may decide they just aren’t going to eat *any* rice,” he says. “And we think the nutritional benefits of eating rice outweigh the risk of exposure to trace amounts of arsenic.”

But simply avoiding rice isn’t feasible for people around the world who rely on the grain as a daily staple. Reducing their intake demands more fundamental changes in how rice is grown and processed—changes that likely won’t be undertaken until regulated standards compel them.
